# Ameliorative Influence of Green Tea Extract on Copper Nanoparticle-Induced Hepatotoxicity in Rats

**DOI:** 10.1186/s11671-015-1068-z

**Published:** 2015-09-16

**Authors:** Marwa A. Ibrahim, A A Khalaf, Mona K. Galal, Hanan A. Ogaly, Azza H.M. Hassan

**Affiliations:** Department of Biochemistry and Chemistry of Nutrition, Faculty of Veterinary Medicine, Cairo University, Giza, Egypt; Department of Forensic Medicine and Toxicology, Faculty of Veterinary Medicine, Cairo University, Giza, Egypt; Department of Pathology, Faculty of Veterinary Medicine, Cairo University, Giza, Egypt

**Keywords:** CNPs, Liver, Apoptosis, Oxidative stress, Green tea extract, Rats

## Abstract

The potential toxicity of copper nanoparticles (CNPs) to the human health and environment remains a critical issue. In the present study, we investigated the protective influence of an aqueous extract of green tea leaves (GTE) against CNPs-induced (20–30 nm) hepatotoxicity. Four different groups of rats were used: group I was the control, group II received CNPs (40 mg/kg BW), group III received CNPs plus GTE, and group IV received GTE alone. We highlighted the hepatoprotective effect of GTE against CNPs toxicity through monitoring the alteration of liver enzyme activity, antioxidant defense mechanism, histopathological alterations, and DNA damage evaluation. The rats that were given CNPs only had a highly significant elevation in liver enzymes, alteration in oxidant-antioxidant balance, and severe pathological changes. In addition, we detected a significant elevation of DNA fragmentation percentage, marked DNA laddering, and significance over expression of both caspase-3 and Bax proteins. The findings for group III clarify the efficacy of GTE as a hepatoprotectant on CNPs through improving the liver enzyme activity, antioxidant status, as well as suppressing DNA fragmentation and the expression of the caspase-3 and Bax proteins. In conclusion, GTE was proved to be a potential hepatoprotective additive as it significantly ameliorates the hepatotoxicity and apoptosis induced by CNPs.

## Background

As more and more nanomaterials are introduced in our daily life, serious environmental hazard could occur. Copper nanoparticles (CNPs) are one of the first engineered nanoparticles (NPs) involved in a variety of industrial applications, such as facial spray, lubricants additive, metallic coating, and inks [1]. Effluent, spillage during shipping, and handling are considered as the main routes of entry for CNPs to human body [2]. Regarding their small size and high reactivity, various studies showed that CNPs could causes a diversity of toxic effects including hepatotoxicity [3, 4]. Chen et al. [4] reported that CNPs’ toxicity is triggered by reactive oxygen species (ROS) over production. Usually, cells respond to oxidative burden through fortifying their antioxidant defense mechanism. However, the imbalance between oxidative burden and defense mechanism induces protein oxidation, lipid peroxidation (LPO), DNA damage, and apoptosis [5, 6]. Recently, much interest had been focused on the role of naturally occurred herbal plant extracts as protective agents for various toxins [7]. Green tea extract (GTE) had attracted a great attention for its health benefits against a variety of toxins associated with oxidative stress [7, 8]. Many studies proved the hepatoprotective role of GTE [9, 10]. The great beneficial influence of GTE was attributed to the high content of catechins. Epicatechin, epicatechin gallate (ECG), epigallocatechin (EGC), and epigallocatechin gallate (EGCG) are the major catechins present in GTE [11]. Those catechins chemically possess multiple hydroxyl substituents responsible for its antioxidant activity [12]. Besides catechins, GTE contains additional antioxidants such as vitamins E and C [13], as well as minerals that function as co-factors for antioxidant enzymes. The discovery of novel protective agents against NPs’ toxicity remains a challenge. Therefore, regarding the mentioned impacts of GTE, as a strong hepatoprotective antioxidant, the present study was designed to evaluate the ameliorative influence of the GTE against CNP-induced hepatotoxicity in male rats.

## Methods

### Animals

Forty-eight male albino rats, weighing 100–120 g, were maintained in stainless steel cages under standard conditions in accordance with the Animal Care and Use Committee of Beni-Suef University. All efforts were made to minimize animal suffering.

### Chemicals

CNPs (20–30 nm), 99.5 % purity, spherical in shape, mineral in nature, were purchased from Sigma Aldrich. A stock suspension of CNPs was prepared by dispersing CNP powder in deionized water followed by vigorous vortexing and sonication [4]. Prior to each use, the stock solution was sonicated for approximately 20 s to ensure proper particle suspension. The green tea was obtained from Lipton green tea Unilever brand, packed in the United Arab Emirates Unilever Gulf FZE. The GTE was prepared according to [14]. Fifteen gram of instant green tea powder was socked in 100 ml of boiling distilled water for 5 min. The solution was filtered to make 1.5 % GTE. The dose of 1.5 % *w*/*v* GTE had been reported as hepatoprotective for rats [15]. The GTE contained EGCG (337 mg/l), EGC (268 mg/l), epicatechin (90 mg/l), ECG (60 mg/l), and caffeic acid (35 mg/l) as determined by the HPLC method [7].

### Experimental Protocol

The rats were equally divided into four different groups. Group I (control) received distilled water only. Group II received CNPs (40 mg/kg BW) via oral gavage. Group III orally received CNPs (40 mg\kg BW) plus GTE (1.5 %, *w*/*v*). Finally, group IV was given GTE (1.5 %, *w*/*v*) alone. The GTE solution was provided to the rats as their sole source of drinking water. The rats were treated for 5 days/week for 2 months. The selected dose of CNPs was 1/10 of the LD50 which was reported by Chen et al. [4]. Throughout the experimental period, there were no signs of toxicity in the animals treated with CNPs detected. At the end of the experiment, the animals were fasted overnight, anesthetized, and sacrificed by cervical dislocation. Blood and liver samples were collected for further estimations.

### Liver Function Tests

The blood samples were collected in plain tubes and centrifuged, and the serum were stored at −20 °C for subsequent serum alanine aminotransferase (ALT), aspartate aminotransferase (AST), and total bilirubin (TB) concentration measurement according to the instructions provided by the manufacturer of the kits.

### Oxidative Stress Parameters

The liver tissue was homogenized in ice-cold 0.1 M phosphate buffer saline (PBS) (pH 7.4) using a Teflon tissue homogenizer. The crude tissue homogenate was centrifuged and used for the measurement of malondialdehyde (MDA) [16], superoxide dismutase (SOD) activity [17], catalase (CAT) activity [18], reduced glutathione concentration (GSH) [19], and total protein concentration [20].

### DNA Fragmentation Assays for Apoptosis

Apoptotic changes in the liver tissue were evaluated by DNA fragmentation percentage using diphenylamine assay (DPA) and DNA laddering assay using agarose gel electrophoresis [21].

### Copper Bioaccumulation in the Liver Tissue

The concentration of copper in the liver tissue was analyzed using an atomic absorption spectrophotometer according to method disrobed by Zheng et al. [22].

### Histopathological Examinations

The liver specimens were fixed in 10 % neutral formalin solution, and dehydrated and embedded in paraffin wax. Blocks were sectioned at a thickness of 5 μm and stained with hematoxylin and eosin [23].

### Immunohistochemical Analysis

For Bax and activated caspase-3 immunostaining, the liver sections were deparaffinized, microwaved, incubated in 3 % H_2_O_2_, and placed in PBS. Blocking of non-specific antibody binding was performed by incubation with normal goat serum at 37 °C. Rabbit anti-caspase-3 (diluted to 1:1000, Abcam, Ltd., USA) and Bax (1:200, Abcam, Ltd., USA) were used as biotinylated primary antibodies. The sections were incubated with peroxidase-conjugated goat anti-rabbit IgG (1:1000). Diaminobenzidine (DAB) was applied as a chromogen to visualize the immune reaction. Images were captured using a digital camera. A hepatocyte with dark brown cytoplasm and nucleus was considered positive cells [24]. Positively immune stained cells were counted in five ×400 magnification fields selected randomly and analyzed according to Yuan et al. [25].

### Statistical Analysis

The data were statistically analyzed using SPSS version 16.0 statistical package. The data are expressed as mean ± SE. Differences between the groups were assessed using one-way analysis of variance (ANOVA). The differences were considered statistically significant for *P* < 0.05.

## Results

### Liver Function Tests

The results revealed that the group II showed a significant elevation in ALT and AST activity and TB concentration compared to group I. Group III protected with GTE showed a significant decrease in ALT by 28.9 %, AST by 27.67 %, and TB by 42.6 % compared to group II whereas non-significant changes were detected compared to the control. Non-significant changes were detected for all parameters between the control and group IV (Table 1).

**Table 1:** The influence of GTE on serum ALT and AST activities and TB concentration in CNP-intoxicated rats

Parameters	Group I	Group II	Group III	Group IV
ALT U/l	35.4 ± 6.42^a^	80.5 ± 5.3^b^	57.2 ± 5.12^ca^	33.4 ± 6.50^da^
AST U/l	73.25 ± 1.2^a^	122.5 ± 1.2^b^	88.7 ± 2.3^ca^	66.6 ± 2.1^da^
TB mg/dl	0.109 ± 0.002^a^	0.521 ± 0.01^b^	0.299 ± 0.032^ca^	0.009 ± 0.005^da^

Data are expressed as mean ± SE for 12 animals per group. *Different superscript letters* within the same row are significantly different

### Oxidative Stress Parameters

**Fig. 1:**
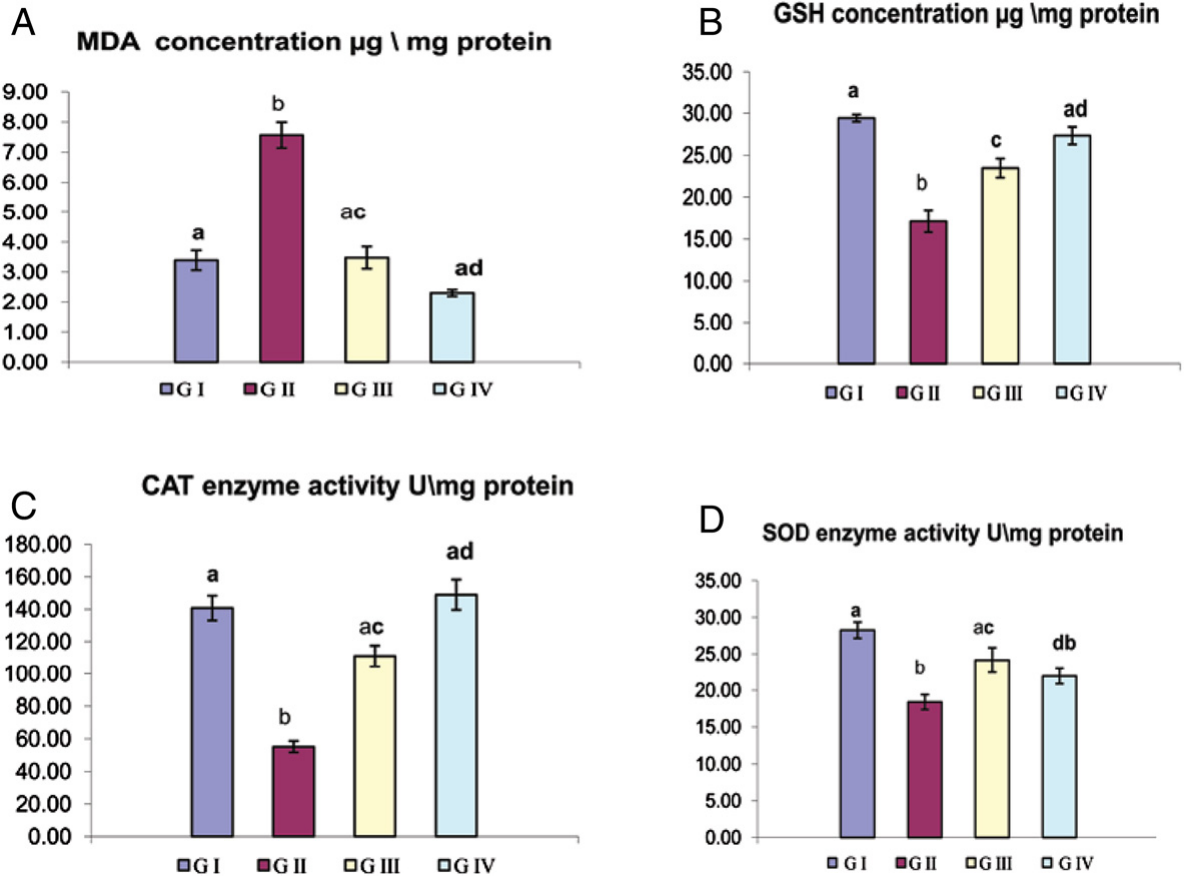
**a**–**d** Influence of GTE on the oxidative stress parameters in liver of CNP-intoxicated rats. Values are expressed as mean ± S.E. *Different superscript letters* are significantly different (*p* < 0.05)*. GI* (group I, control), *GII* (group II treated with CNPs), *GIII* (group III treated with CNPs plus GTE), and *GIV* (group IV treated with GTE)

It was obvious that MDA, the indicative marker for LPO, showed a significant elevation in group II (7.56 ± 0. 43) compared to group I (3.39 ± 0.33). Administration of GTE in group III caused significant reduction in the elevated MDA by 53.9 %. Under normal condition, the over ROS production were neutralized by the antioxidant defense mechanisms. GSH is an important non-enzymatic antioxidant that plays a crucial role in the detoxification of ROS. SOD and CAT enzymes are the first line of cellular defense against oxidative injury. In the current study, the oral administration of CNPs for group II led to a significant reduction in GSH (from 29.39 ± 0.43 to 17.12 ± 1.3), CAT (from 140.7 ± 7.6 to 55.2 ± 6.5), and SOD (from 28.25 ± 3.1 to 18.37 ± 1.03) activities compared to the control. Co-administration of GTE to group III caused a significant increase in GSH by 37.1 % and both enzymes activities CAT by 101.1 % and SOD by 31.4 % which nearly returned to its normal values when compared to group I. Both groups I and IV showed non-significant differences among all oxidative stress parameters except SOD (Fig. 1).

### DNA Damage Assay

**Fig. 2:**
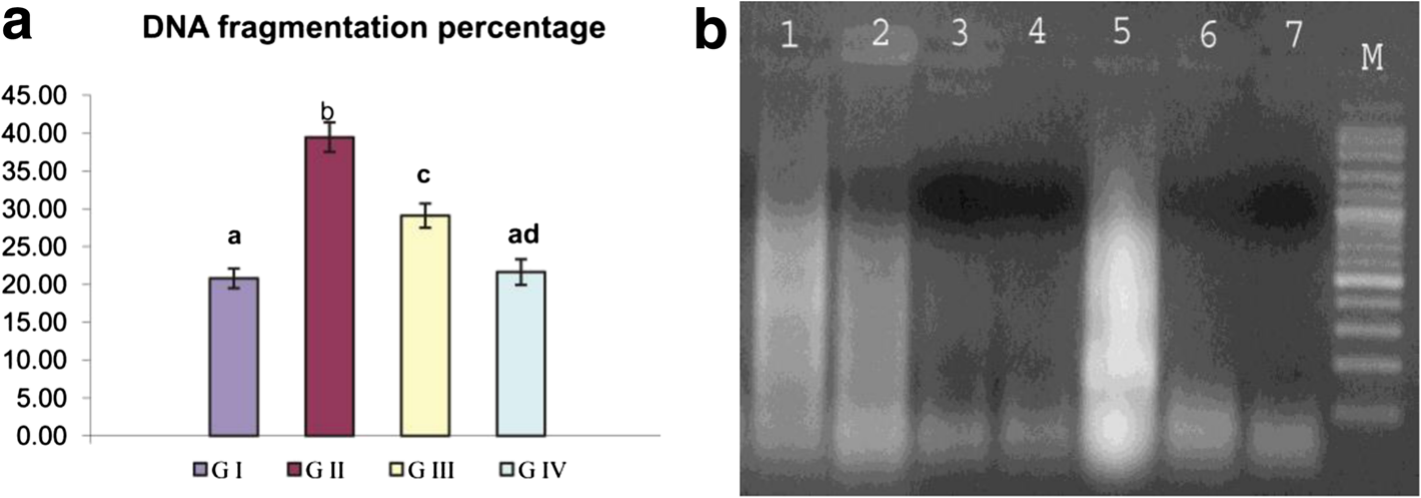
**a** Influence of GTE on DNA fragmentation percentage in the liver of CNP-intoxicated rats. Values are expressed as mean ± SE. *Different superscript letters* are significantly different (*p* < 0.05)*. GI* (group I, control), *GII* (group II treated with CNPs), *GIII* (group III treated with CNPs plus GTE), and *GIV* (group IV treated with GTE). **b** Agarose gel electrophoresis for the fragmented DNA from the liver tissue. *Lanes 1* and *2* showed smear patterns for group III, *lanes 3* and *4* showed lack of DNA laddering for group I, *lane 5* marked DNA ladder in group II, and *lanes 6* and *7* showed lack of DNA laddering for group IV. *M* 100-bp DNA marker

DNA fragmentation is a very typical feature for apoptosis. Both quantitative and qualitative DNA fragmentation in hepatic tissue were evaluated in the current study (Fig. 2). CNPs caused marked elevation in DNA fragmentation percentage (39.48 ± 1.9) in group II compared to group I (20.79 ± 1.3). Oral administration of GTE for group III caused significant reduction in DNA fragmentation percentage by 26.2 %. There are non-significant changes between group I and IV detected. Marked DNA laddering induced by CNPs was observed in group II compared to group I. GTE administration for group III showed a marked decrease in DNA laddering. Lacking of DNA laddering was observed in both groups I and IV.

### Copper Bioaccumulation in the Liver Tissue

**Fig. 3:**
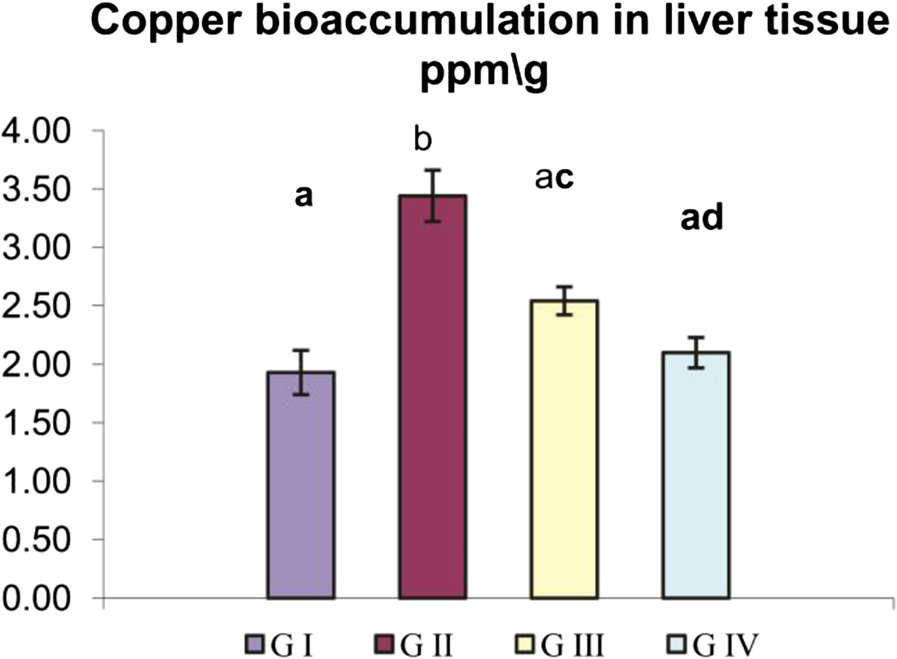
Influence of oral administration of GTE on copper bioaccumulation in the liver of CNP-intoxicated rats. Values are expressed as mean ± SE. *Different superscript letters* are significantly different (*p* < 0.05)*. GI* (group I, control), *GII* (group II treated with CNPs), *GIII* (group III treated with CNPs plus GTE), and *GIV* (group IV treated with GTE)

According to our study, a significant elevation in copper accumulation was observed in group II intoxicated with CNPs (3.44 ± 0.22) compared to the control (1.93 ± 0.19). The protective group III treated with GTE showed a significant reduction in the copper accumulation by 26.1 %. No significant differences were detected between the group I and group IV (Fig. 3).

### Histopathological Analysis

**Fig. 4:**
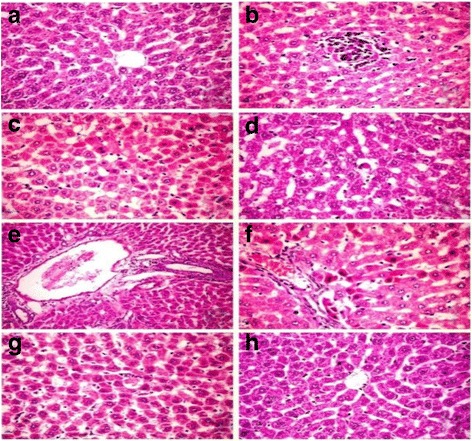
Photomicrograph of sections in rat liver intoxicated with CNPs (H and E ×400). **a** Group I showing normal hepatic parenchyma. Group II showing **b** focal area of hepatocellular necrosis infiltrated by mononuclear cells, **c** polyploidy hepatocytes represented by hepatic cytokaryomegaly, binucleated hepatocytes associated with activation of Kupffer cells and sporadic cell necrosis **d** apoptosis, **e** oval cell proliferation,hyperplasia of biliary epithelium and formation of newly formed bile ductule and **f** periportal sporadic hepaticcell necrosis. **g** Group III showing individual cell necrosis. **h** Group IV showing normal hepatic parenchyma

The liver of group I showed normal hepatic parenchyma with no evidence of hepatocellular necrosis or inflammatory reaction (Fig. 4a). Meanwhile, the liver of group II revealed various histopathological alterations characterized by focal area of hepatocellular necrosis infiltrated by mononuclear cells (Fig. 4b) and polyploidy hepatocytes represented by hepatic cytokaryomegaly, binucleated hepatocytes associated with activation of Kupffer cells and sporadic cell necrosis (Fig. 4c) as well as apoptosis (Fig. 4d). Portal triad revealed oval cell proliferation, hyperplasia of biliary epithelium, and formation of newly formed bile ductules (Fig. 4e) in addition to periportal sporadic hepatic cell necrosis and apoptosis (Fig. 4f). These histopathological alterations were markedly reduced in the group III treated with GTE as the liver showed mild granular degeneration of hepatocytes and individual cell necrosis (Fig. 4g). The liver of group IV showed nearly similar picture to those demonstrated in the control one (Fig. 4h).

### Immunohistochemical Analysis

**Fig. 5:**
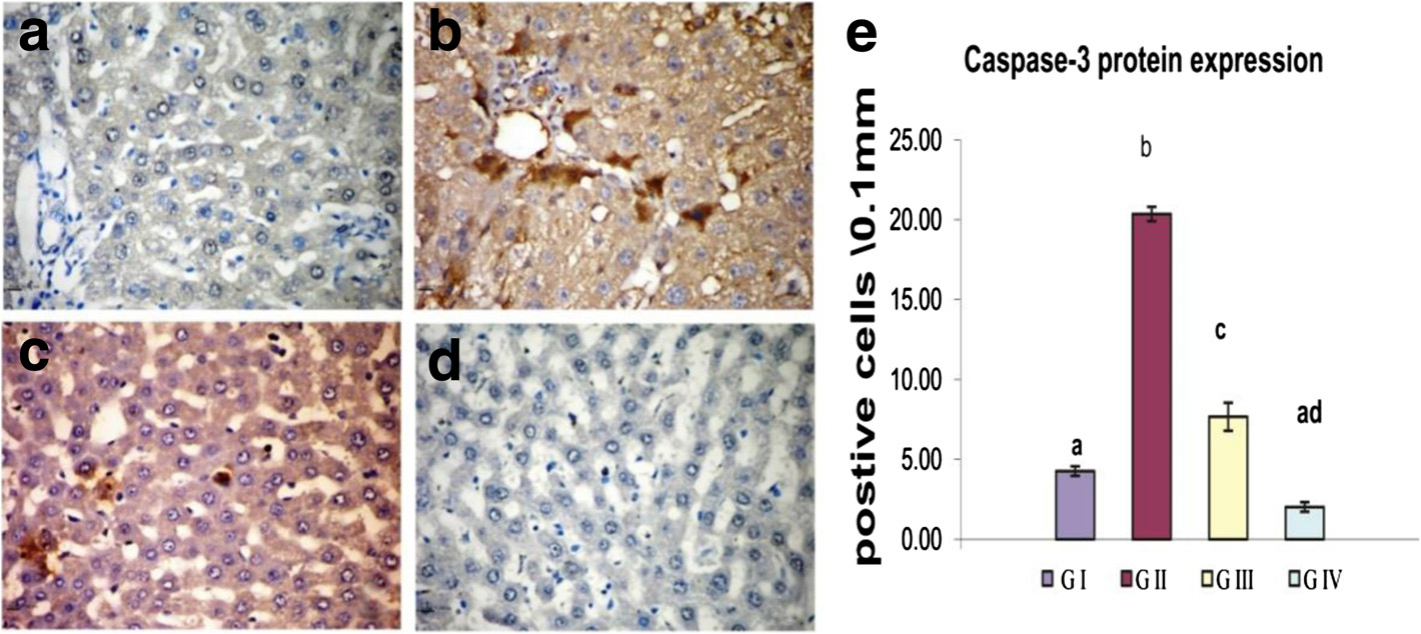
Influence of oral administration of GTE on caspase-3 protein expression in the liver of CNP-intoxicated rats. **a** Group I and **d** group IV showed negatively immune stained cells for caspase-3 protein expression. Group II (**b**) showing marked elevation for the positively immune stained cells. Group III (**c**) showing reduced number of positively stained cell. **e** The *bar chart* represents the relative number of immune positive cells for caspase-3 protein in liver tissue of different groups. Values are expressed as mean ± SE. *Different superscript letters* are significantly different (*p* < 0.05)*. GI* (group I, control), *GII* (group II treated with CNPs), *GIII* (group III treated with CNPs plus GTE), and *GIV* (group IV treated with GTE)

**Fig. 6:**
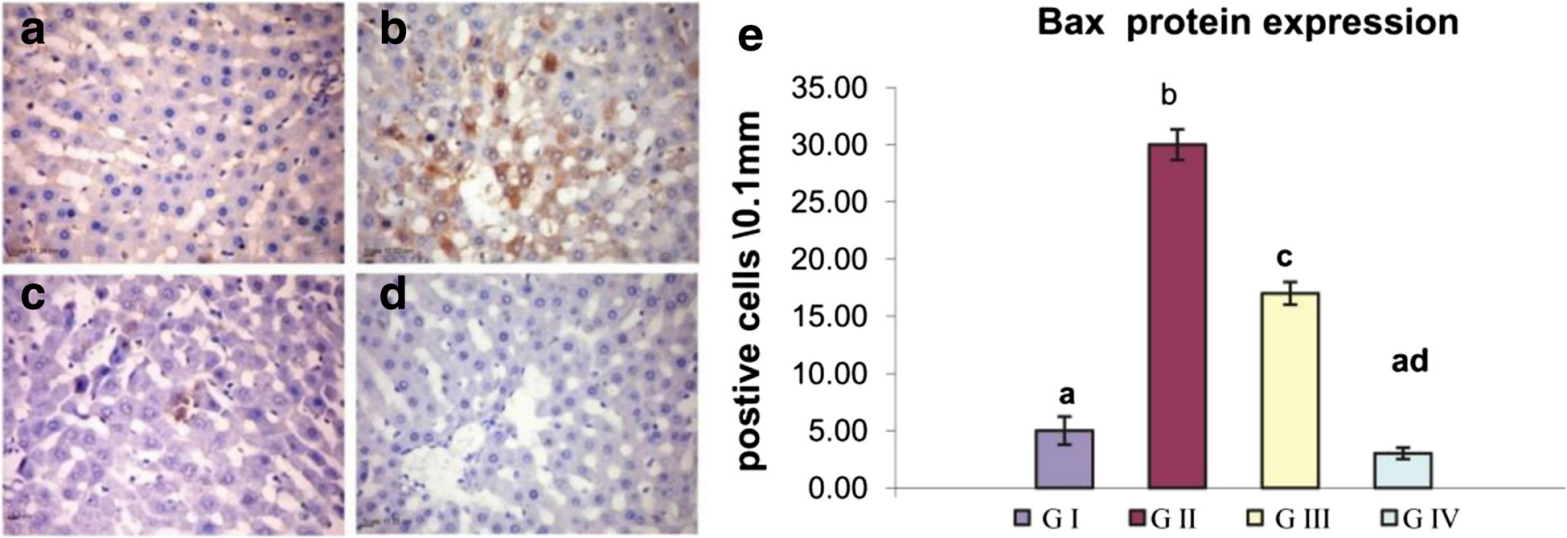
Influence of oral administration of GTE on Bax protein expression in the liver of CNP-intoxicated rats. **a** Group I and **d** group IV showing negatively immune stained cells for Bax protein. Group II (**b**) showing greater number of positively immune stained cells. Group III (**c**) showing moderate number of positively stained cell. **e** The *bar chart* represents the relative number of immune positive cells for Bax protein in the liver tissue of the different groups. Values are expressed as mean ± SE. *Different superscript letters* are significantly different (*p* < 0.05)*. GI* (group I, control), *GII* (group II treated with CNPs), *GIII* (group III treated with CNPs plus GTE), and *GIV* (group IV treated with GTE)

Figures 5 and 6 summarized the results of immunohistochemical evaluation of caspase-3 and Bax proteins expression in the different experimental groups. A significant elevation in caspase-3 immuno-positive hepatocytes was detected in group II (20.35 ± 0.46) compared to group I (4.25 ± 0.30). The number of caspase-3 immunopositive cells was significantly reduced in response to GTE administration in group III (Fig. 5). Similarly, the Bax protein expression showed a significant increase in group II as indicated with elevation of immune reactive hepatocytes (30.2 ± 1.34) compared to group I (5.32 ± 1.23). Reduction of Bax protein expression by GTE co-administration was clearly observed in group III (17.4 ± 0.99). Non-significant changed in caspase-3 and Bax immunostaining were detected in group IV compared to the control.

## Discussion

Manufactured NPs and their applications are expanding in the fields of technology. With massive introduction of these materials in our life, it was important to investigate their possible adverse effects on human health. The small size and large surface area of NPs were the main reason attributed for ROS over production which is the main mechanism of NPs’ toxicity [4]. Excessive production of ROS plays a crucial role in the induction and progression of several diseases such as the liver [26]. In the present investigation, we highlighted on the hepatoprotective influences of GTE on CNP-induced apoptotic effects triggered over production of ROS. The liver tissue is a critical organ for metal storage, metabolism, and detoxification [27]. According to our results reported in Table 1, we observed a significant elevation in ALT and AST enzyme activity and TB level, the efficient indicators for liver damage, in group II. In the liver injury, the transport function of hepatocytes is disturbed, resulting in leakage of plasma membrane and elevation of the serum level of liver enzymes [28, 29]. The increased TB concentration might be attributed to the failure of normal uptake, conjugation and excretion by the damaged hepatic parenchyma [30]. Recent experimental studies had shed a new light on the chemical and biological aspects of ROS and its role in pathogenesis of many diseases. Transition metals including copper are involved in ROS generation via mechanism of Fenton-type reaction in which the metal ion reacts with H_2_O_2_ to yield hydroxyl radical that is extremely reactive and toxic to biological molecules in addition to the oxidized metal ion itself [31]. In the same sequence, CNPs consumed hydrogen ions in the stomach at a faster rate and converted into cupric ions with higher toxicity [32]. Once CNPs gain access into the mitochondria, they stimulate ROS production via impairment of electron transport chain, activation of NADPH-like enzyme system, and damage to membrane phospholipids inducing membrane depolarization [33]. In the present study, the significant elevation in liver MDA and reduction of GSH level, SOD and CAT enzyme activity in group II (Fig. 1) was widely accepted sign of oxidative stress. When the production of ROS exceeds the capacity of cellular antioxidant machineries, accumulation of pro-oxidants occurred leading to a state of oxidative stress [34]. Our data represented in Fig. 1 are consistent with recently published data demonstrating that CNPs were able to generate oxidative damage [35, 36]. The metal NPs are mainly accumulated in the liver regardless of their size, shape, dose, and types of materials. According to our results detected in Fig. 3, the oral administration of CNPs to group II led to significant accumulation of copper in the liver tissue, our results are in the same consequence with the data reported by Privalova et al. [37]. The greater bioaccumulation of CNPs in the liver may be attributed to the fenestrated, discontinuous endothelia of the liver which allow the passage of NPs up to 100 nm from the blood into the liver parenchyma. In addition, liver can efficiently accumulate high amounts of NPs via opsonization [38]. Previous studies indicate that copper can be metabolized in hepatic tissue and be transferred to metallothionein by GSH thus, the copper overload is reached and depletion of GSH instantaneously results in enhanced cellular toxicity [39]. Excess ROS production could damage hepatocytes and activate hepatic satellite cells, which play a central role in liver damage and fibrosis [40]. Although apoptosis is an essential process for development of multicellular organisms, its abnormal induction could motivate various diseases [41]. NPs are able to induce mitochondrial damage through the direct interaction with undissolved NPs following endocytotic uptake and/or ROS-derived LPO with disruption of the membrane integrity and release of apoptotic enzymes [42]. To monitor the ability of GTE to counteract the apoptotic effects induced by CNPs, DNA fragmentation percentage, DNA laddering assay, and the expression of some apoptotic genes were evaluated. DNA fragmentation is a very ideal form for the apoptotic process with generation of multiples fragments through the action of endonuclease. According to our study, higher DNA fragmentation percentage and marked DNA laddering (Fig. 2) were detected in group II. Findings from several studies were in the same consequence with our results [36, 43]. Excessive ROS production induced by CNPs caused oxidative damage to single base and sugar phosphate of DNA and breaks DNA strands [44]. In addition, Cu^2+^ decreases cell viability by binding to DNA resulting in cell death [45]. Apoptosis is tightly regulated by the expression or activation of several genes and proteins [46]. Accumulating evidences have indicated that NPs could induce a cellular apoptosis by targeting the mitochondrial apoptotic pathway, through activation cytochrome c release from the mitochondria, decreasing Bcl-2 protein expression over expression of Bax, translocation of Bax into mitochondrial membrane, and activation of caspase-3 activity [47, 48]. Caspase activation plays a central role in the execution of apoptosis. According to our results, a significant increase of caspase-3 and Bax proteins expression in group II were detected (Figs. 5 and 6). Our finding is supported by other previous studies that reported that CNPs induced apoptosis directly through the alteration of apoptotic genes expression [4, 35]. All those changes occurred on the molecular level were confirmed by histopathological analysis as observed in Fig. 4. These alterations could be attributed to the cyto- and genotoxic effects of CNPs correlated with higher copper accumulation in the hepatocytes. Those histopathological observations were in quite agreement with the results reported by [49]. Several epidemiologic data showed that special dietary additives could provide effective defenses against oxidative stress and thus have potential as protective and or treatment for a variety of diseases. Since ancient times, green tea consumption is considered as nature’s gift for promoting human health. The present study demonstrates that GTE offered partial hepatic protection through reduction of serum ALT and AST enzyme activity and TB (Table 1), as well as restoring the antioxidant enzymes (SOD, CAT) activity and GSH concentration (Fig. 1), reduction of MDA level (Fig. 1a), minimization of DNA fragmentation percentage, DNA laddering, and downregulation of some apoptotic genes caspase-3 (Fig. 5e) and Bax protein expression (Fig. 6e). All these observations were supported by the healing view of hepatic parenchyma and regeneration of hepatocytes observed through the histological analysis (Fig. 4). The underlying mechanisms attributed to the hepatoprotective influence of GTE against CNPs could be contributed to the following: the gallate groups of catechins (EGCG, ECG, and EGC) present in GTE are thought to inhibit the Fenton-like reaction [50], those catechins act as a powerful hydrogen-donating radical scavenger, thus, the formation of highly reactive hydroxyl radicals (OH·) were inhibited and this in turn, could prevent LPO; the catechins present in GTE chelate divalent transition metal ions via their ortho-hydroxy-phenolic groups, preventing the CNP-induced formation of free radicals by restricting the access of the metal ion toward lipid biomembranes [51]; EGCG and other catechins induce mild level of oxidative stress which may lead to the induction of expression of intracellular endogenous antioxidants [52]; in addition, the catechins exert their antioxidant and anti-apoptotic effects through the ultra rapid electron transfer from catechins to ROS-induced radical sites on DNA molecules [53]. Pan et al. [54] reported that EGCG could induce the activation of intracellular signaling cascades such as the mitogen-activated kinase pathway and phosphoinositol-3-kinase pathway which had potent roles in anti-apoptotic signaling. In the same line, EGCG and its methylated metabolite protect against necrosis and apoptosis by suppressing caspase-3 expression, reducing the expression of proapoptotic genes and inducing the anti-apoptotic genes expression [55, 56]. Tea catechins, especially EGCG and EGC, reduced the cytotoxicity by suppressing the cytochrome c release from mitochondria to cytosol and subsequent caspase activation [57]. Catechins also attenuates the expression of α-smooth muscle actin (α-SMA) which had crucial roles in the pathogenesis of tissue fibrosis through inhibiting the signal transduction of TGF-β binding to its receptors [58]; GTE contains also minerals that function as co-factors for antioxidant enzymes. Zinc traces present in GTE considered as a selective inhibitor of apoptosis [59]. The hepatoprotective effect of GTE against liver dysfunction was reported by Safer et al. [60] and Gad and Zaghloul [9]. Numerous studies based on in vivo and in vitro study confirmed that GTE and their constituents show health-promoting protective effect at a certain dose concentration [61]. Low to moderate doses of GTE or EGCG have reported no serious adverse effects [62]. On the other hand, GTE or EGCG overdose causes adverse complications including liver failure and hepatotoxicity [63]. An experiment based on rat model revealed that oral dose of 2000 mg EGCG/kg was lethal but dose with 200 mg EGCG/kg induced no toxicity [64]. The GTE was more stable than pure ECGC because of the presence of other antioxidant constituents in the extract [65]. In general, herbal medicines are complex mixtures of different compounds that act synergistically and exert their full beneficial effect as total extracts [66]. This is the cause why we choose to work on the natural extracts and not one of its ingredients.

## Conclusions

Excessive accumulation of CNPs in the liver caused several adverse effects including changes in liver enzyme activities, generation of ROS, marked pathological changes, DNA damage, and apoptosis. Based on our results, we propose that GTE could provide a cushion for prolonged protective benefit against CNP-induced hepatotoxicity without harmful side effects through its potent antioxidant and antiapoptotic properties.

## Abbreviations

CNPs: copper nanoparticles
ECG: epicatechin gallate
EGC: epigallocatechin
EGCG: epigallocatechin gallate
GTE: green tea extract
LPO: lipid peroxidation
NPs: nanoparticles
ROS: reactive oxygen species
